# Synthesis, crystal structure and Hirshfeld surface analysis of bis­(1*H*-benzimidazole-κ*N*^3^)bis(benzimidazole-2-carboxyl­ato-κ^2^*N*^3^,*O*)cobalt(II)

**DOI:** 10.1107/S2056989025011211

**Published:** 2026-01-01

**Authors:** Farangiz Khujayeva, Sardor Murodov, Rukhshona Muratkulova, Soliha Rixsiboyeva, Kambarali Turgunov, Bakhodir Tashkhodjaev, Shakhlo Daminova

**Affiliations:** a National University of Uzbekistan named after Mirzo Ulugbek University Street 4 Tashkent 100174 Uzbekistan; b Uzbekistan-Japan Innovation Centre of Youth, University Street 2B, Tashkent, 100095, Uzbekistan; c Tashkent Pharmaceutical Institute, A. Aybek Street, 45, Tashkent 100015, Uzbekistan; d Institute of the Chemistry of Plant Substances, Uzbekistan Academy of Sciences, Mirzo Ulugbek Str. 77, Tashkent 100170, Uzbekistan; Tokyo University of Science, Japan

**Keywords:** crystal structure, hydrogen bond, Hirshfeld surface, benzimidazole, benzimidazole-2-carb­oxy­lic acid

## Abstract

The title complex, [Co(C_8_H_5_N_2_O_2_)_2_(C_7_H_6_N_2_)_2_], crystallizes in the monoclinic space group *P2*_1_*/c* with one-half of the mol­ecule in the asymmetric unit. The Co^2+^ ion exhibits a distorted octa­hedral environment formed by two monodentate benzimidazole ligands and two bidentate benzimidazole-2-carboxyl­ate ligands. The crystal packing features N—H⋯O hydrogen bonds and C—H⋯π contacts, which generate a chain along [011]. Hirshfeld surface analysis shows that H⋯C/C⋯H (36.2%) and H⋯H (35.3%) contacts dominate the inter­molecular inter­actions, followed by O⋯H/H⋯O and N⋯H/H⋯N contributions.

## Abstract 2

The cobalt(II) complex, [C_30_H_22_CoN_8_O_4_], crystallizes in the monoclinic space group *P2*_1_*/c*, with the Co^+2^ ion adopting an octa­hedral coordination environment. The crystal structure features N—H⋯O hydrogen bonds and C—H⋯π contacts forming a chain along [011]. Hirshfeld surface analysis indicates that H⋯C/C⋯H and H⋯H contacts dominate the inter­molecular inter­actions.

## 1. Chemical context

Benzimidazole is an aromatic heterocyclic ligand containing two nitro­gen atoms in a five-membered fragment: one can serve as a coordination donor, while the second bears a proton (N—H) and is capable of forming directional hydrogen bonds, which determines its behavior in complexation (Walia *et al.*, 2011 ▸). Thanks to its delocalized π-system, benzimidazole is prone to π–π and C—H⋯π inter­actions, which significantly influence crystal packing and the inter­molecular interactions in complexes (Keri *et al.*, 2015 ▸). Electron-donating or -accepting substituents on the benzene or imidazole ring markedly alter the nitrogen donor character, enabling tuning of the metal-center electron density, stabilization of particular oxidation states and modification of the coordination geometry (Bansal & Silakari, 2012 ▸). Benzimidazole derivatives demonstrate a wide spectrum of biological activities and are therefore frequently used in the development of bioactive mol­ecules and pharmaceutical candidates (Singh *et al.*, 2012 ▸). Taken together, these features make benzimidazole a versatile and readily modifiable building block for the synthesis of functional coordination complexes.

Benzimidazole-2-carb­oxy­lic acid is a benzimidazole functionalized at the 2-position with a carboxyl group, which imparts additional acidic and coordination activity to the mol­ecule. Classical methods for its preparation and reactions are described in detail by Copeland & Day (1943 ▸). Derivatives of benzimidazole-2-carb­oxy­lic acid exhibit pharmacological activity, including anti-inflammatory effects, and their synthetic modifications have been explored to obtain bioactive compounds (Thakurdesai *et al.*, 2007 ▸). The carboxyl function enables the formation of carboxyl­ate coordination and bridging between metals, as well as directional O—H⋯*A* and N—H⋯O hydrogen bonds, which substanti­ally affect crystal packing, complex stabilization, and their chemical properties (Doring *et al.*, 1997 ▸).

In this context, we synthesized the title complex [Co(C_8_H_5_N_2_O_2_)_2_(C_7_H_6_N_2_)_2_], (**I**). This work provides an analysis of the structural and supra­molecular properties and of the Hirshfeld surface.
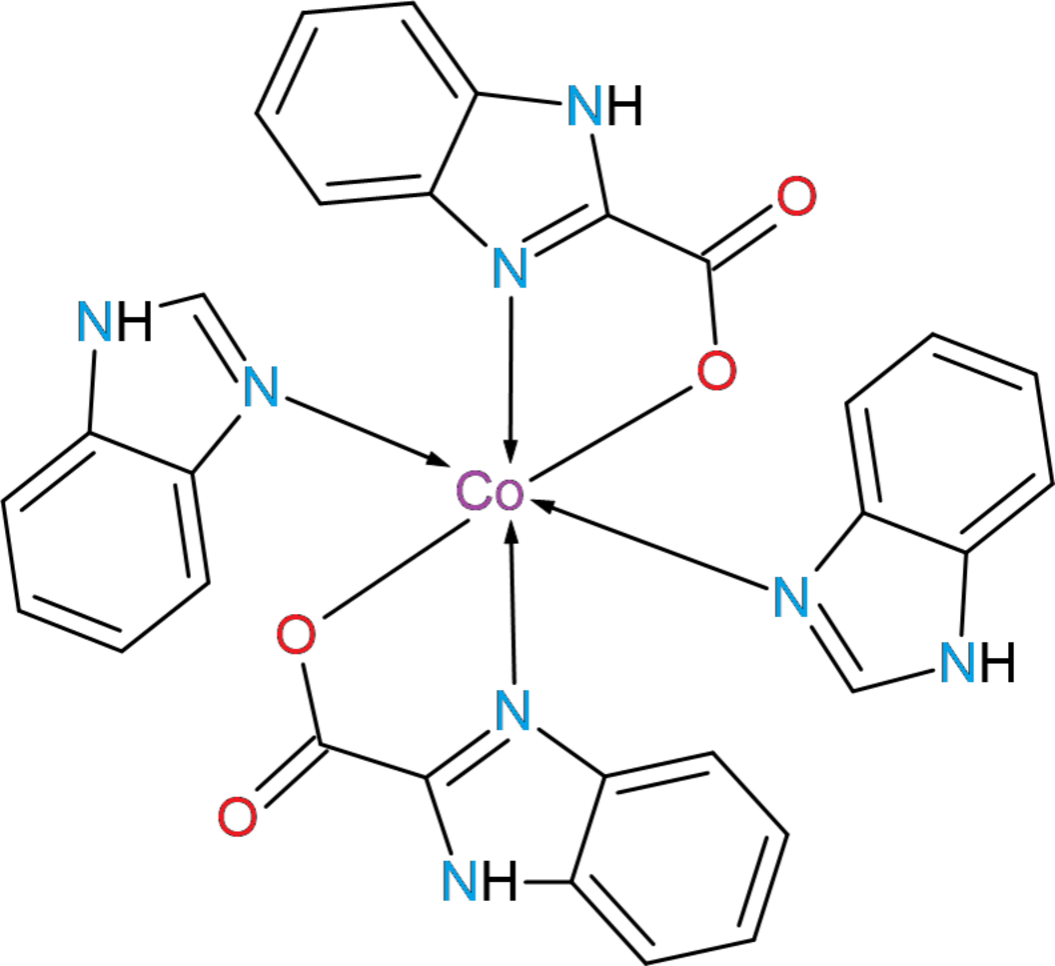


## 2. Structural commentary

**Figure 1:**
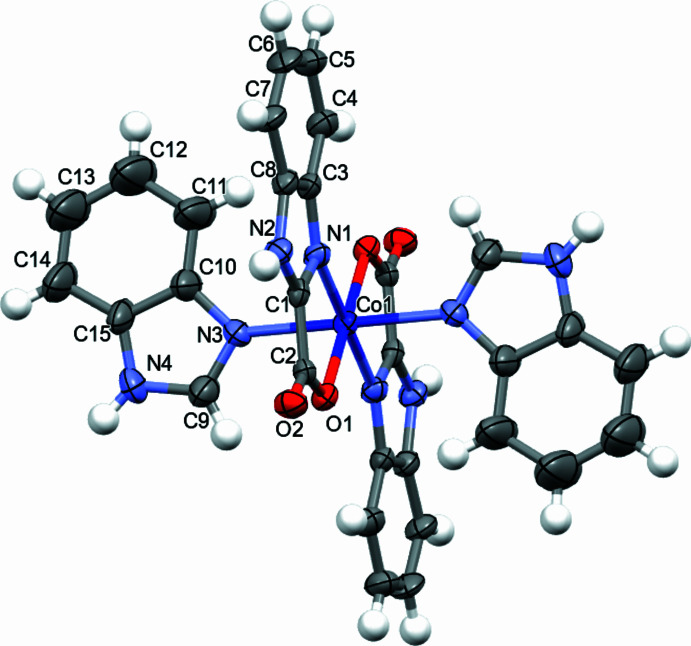
Molecular structure of the title compound with displacement ellipsoids drawn at the 30% probability level. Only atoms of the asymmetric unit are labelled; the remaining atoms are generated by the symmetry operation −*x*, −*y*, −*;z*.

The structure of (**I**) crystallized in space group *P2*_1_*/c* with half a molecule in the asymmetric unit (Fig. 1 ▸). The central Co^II^ atom (site symmetry 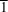) is coordinated by four ligands and has a coordination number of six; two of these are neutral benzimidazole molecules (BI), coordinated monodentately through an *sp*^2^ nitro­gen atom [Co—N3 = 2.162 (5) Å]. The other two ligands are benzimidazole-2-carb­oxy­late anions (BICA), which acts as a bidentate ligand and coordinates *via* the carboxyl­ate oxygen atom [Co—O1 = 2.128 (4) Å] and the *sp*^2^ nitro­gen atom [Co—N1 = 2.101 (5) Å] of the imidazole ring (Table 1 ▸). The BICA ligand forms a five-membered chelate ring, with a chelate angle N1—Co1—O1 = 79.00 (18)° (Table 1 ▸). The coordination environment around the central Co^2+^ ion is distorted n octa­hedron (4 + 2), with four shorter equatorial Co—N/O bonds and two longer axial Co—N/O bonds. The metal–ligand distances range from 2.101 (5) to 2.162 (5) Å, which is consistent with a Co^2+^ oxidation state, since Co^3+^ typically exhibits shorter bond lengths (≈1.8–2.0 Å), especially to oxygen atoms (Heffern *et al.*, 2015 ▸; Tojiboyeva *et al.*, 2025 ▸). The bond-valence sum calculated for Co1 is 1.94 v.u., further supporting the assignment of the +2 oxidation state. The BI and BICA ligands are not coplanar: the dihedral angle between their least-squares (mean) planes in the asymmetric unit is 81.7(4)°, a pronounced non-coplan­arity that may influence the molecular geometry and crystal packing.

Structural data for the obtained complex were compared with a related synthesized complex (Döring *et al.*, 1997 ▸) in which the coordination environment includes benzimidazole and quinaldinate ligands. The cobalt atom in that structure also lies on an inversion centre but the five-membered chelate ring formed by the quinaldinate ligand has a bite angle of 77.52°, which is 1.5° smaller than in the present complex. This difference can be attributed to ligand flexibility, stereochemical factors (including variations in the chelate-ring conformation, metal–ligand bond distances and the relative orientation of donor atoms), as well as crystal packing effects. Axial bond lengths reported for that complex are Co—N1 = 2.174 (3) Å for the two benzimidazole ligands, compared with Co—N3 = 2.162 (5) Å in our complex. Equatorial bond lengths in the referenced complex are Co—O1 = 2.051 (2) Å and Co—N3 = 2.218 (2) Å, whereas the corresponding values in our complex are 2.128 (4) and 2.101 (5) Å, respectively. Such pronounced differences in the equatorial plane most likely reflect variations in the donor strengths and conformations of the respective ligands (different electron densities on the O and N donors), as well as the influence of the bite angle and crystal-packing effects (hydrogen bonds and C—H⋯π contacts), which can further ‘stretch’ or ‘compress’ individual bonds.

## 3. Supra­molecular features

**Figure 2:**
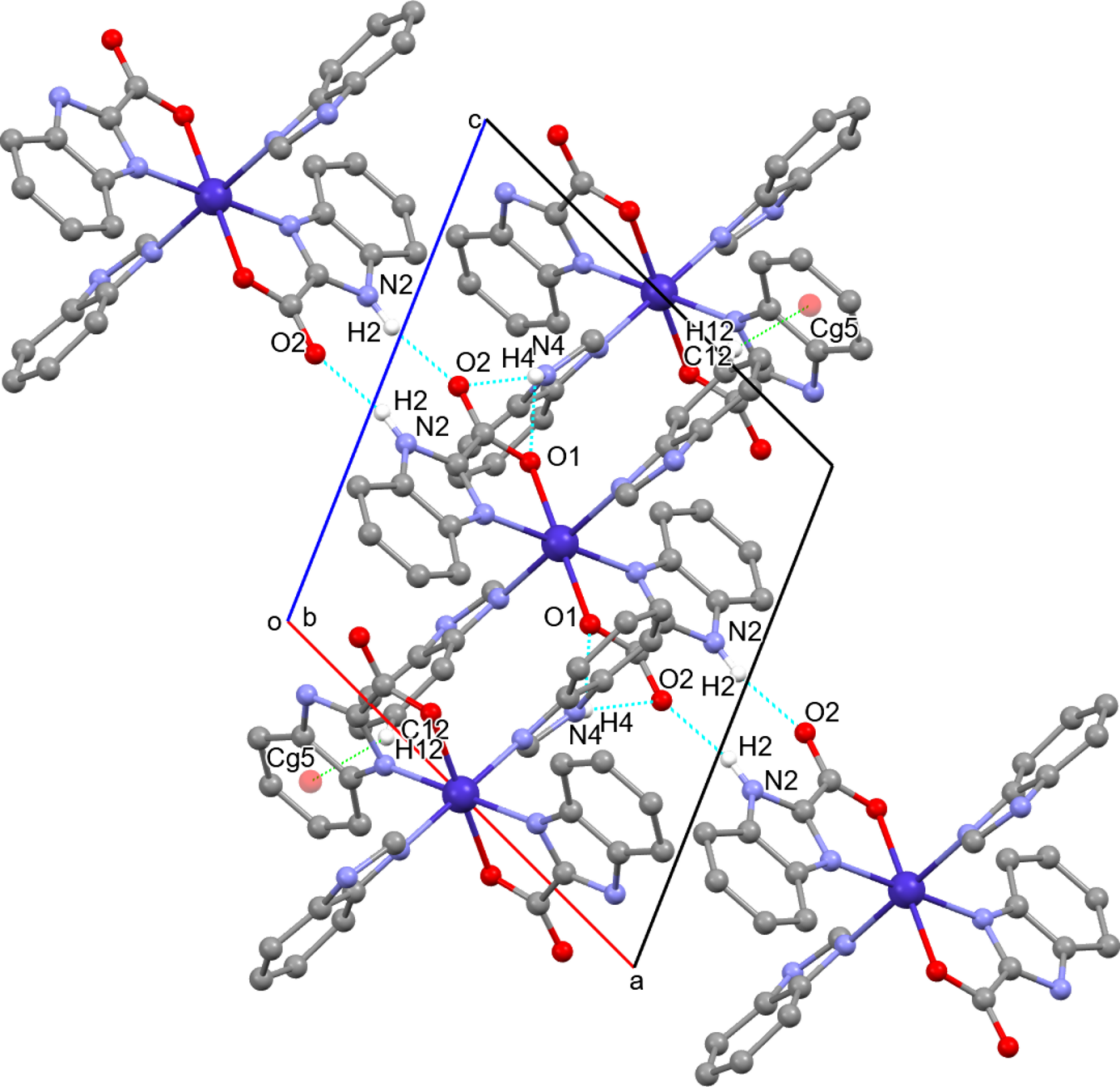
Packing diagram for (**I**) illustrating the inter­molecular hydrogen-bonding: classical N—H⋯O and non-classical C—H⋯π inter­actions. Classical N—H⋯O hydrogen bonds are shown as blue dashed lines; non-classical C—H⋯π inter­actions are shown in green. These contacts form chains along the [011] direction.

In the crystal, pairs of N—H⋯O hydrogen bonds link adjacent BICA ligands: N2—H2⋯O2^ii^ [symmetry code: (ii) −*x*, −*y* + 1, −*z* + 1] and N4—H4⋯O1^iii^ [and the bifurcated contact N4—H4⋯O2^iii^; symmetry code: (iii) −*x* + 1, *y* − 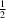, −*z* + 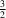]. These pairs connect mol­ecules into a chain running along the crystallographic [100] direction (Fig. 2 ▸*a*). In addition to the classical N—H⋯O inter­actions, a C—H⋯π contact [C12—H12⋯*Cg*5^iv^; symmetry code: (iv) −*x* + 1, *y* + 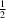, −*z* + 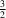] is also identified, reinforcing the packing and providing additional cohesion to the supra­molecular network (Fig. 2 ▸*b*). The hydrogen bonds and non-classical contacts form a directional chain along [011]; all inter­molecular contacts are presented in Table 2 ▸.

## 4. Hirshfeld surface

A Hirshfeld surface analysis was performed using *CrystalExplorer 21.5* (Spackman *et al.*, 2021 ▸) and *d*_norm_ maps and two-dimensional fingerprint plots were generated, providing a qu­anti­tative assessment of the contributions of different types of inter­molecular contacts to the total Hirshfeld surface.

**Figure 3:**
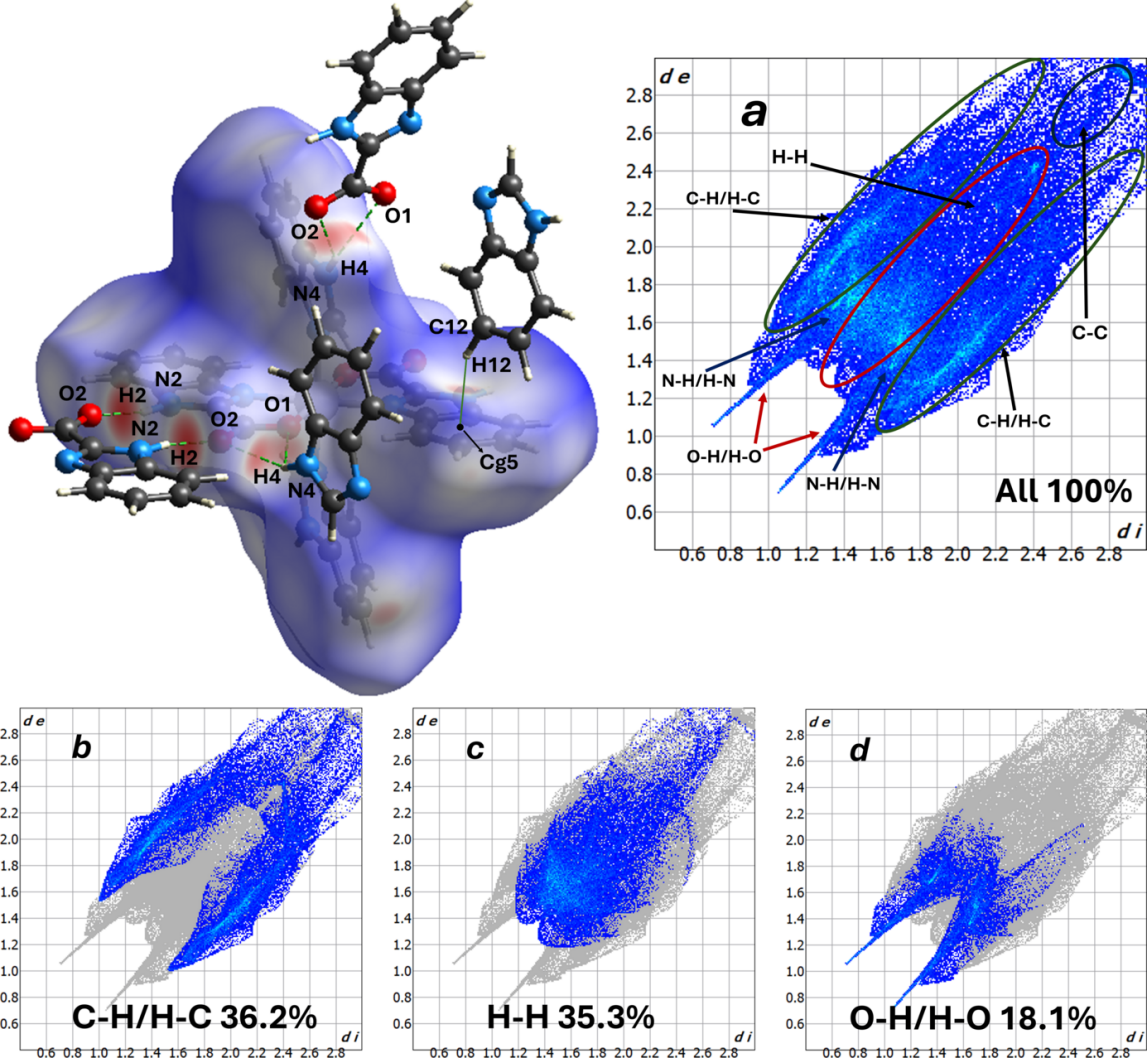
Two-dimensional fingerprint plots for the title compound, showing (*a*) all inter­actions, and decomposed into (*b*) C⋯H/H⋯C, (*c*) H⋯H and (*d*) O⋯H/H⋯O inter­actions.

In the *d*_norm_ map (Fig. 3 ▸), localized dark-red spots correspond to contacts shorter than the sum of the van der Waals radii, white regions indicate contacts close to the sum of the radii, and blue areas denote elongated contacts. In the complex under study, the most significant close contacts and directional inter­molecular inter­actions are represented by the following inter­actions identified in the structural analysis. The pronounced red spots around atoms N2/N4 and O1/O2 indicate directional N—H⋯O hydrogen bonds. The presence of small red spots in the region of the aromatic ring points to possible π–π and C—H⋯π contacts. These contacts are consistent with the localized red spots on the *d*_norm_ map, indicating the presence of directional hydrogen bonds and edge contacts between aromatic fragments.

The two-dimensional fingerprint plots (Fig. 4) qu­anti­tatively characterize the contribution of different contact pairs to the overall Hirshfeld surface. For this structure the following contributions are observed: H⋯C/C⋯H = 36.2%, H⋯H = 35.3%, O⋯H/H⋯O = 18.1%, N⋯H/H⋯N = 7.3%, C⋯C = 2.6% and N⋯C/C⋯N = 0.5%. The approximately equal contributions of H⋯C and H⋯H contacts indicate that mol­ecular packing is determined both by close van der Waals inter­actions between hydrogen atoms and by numerous edge contacts between aromatic rings (C—H⋯π and close ring–ring approaches). The significant contribution of O⋯H/H⋯O (18.1%), together with the notable contribution of N⋯H/H⋯N (7.3%), confirms the presence of directional hydrogen bonds in the crystal, which include N—H⋯O-type contacts, which play an important role in consolidating the packing. The low C⋯C contribution (2.6%) indicates that direct π–π inter­actions between rings are present but are not a dominating factor – this is consistent with the visual data from the *d*_norm_ map.

## 5. Database survey

A search of the Cambridge Structural Database (CSD, version 2024.2.0; Groom *et al.*, 2016 ▸) showed that no similar structures containing a cobalt–BICA fragment were recorded. A search for a cobalt–BI fragment yielded 1053 similar structures, among which one can find related compounds, for example a cobalt complex based on benzimidazole and quinaldinate (CSD refcode BECFAR; Döring *et al.*, 1997 ▸). In addition, cobalt coordination compounds based on benzimidazole with various secondary ligands can be found [CSD refcodes YOJJAK (Cheng *et al.*, 2013 ▸), BAWNAP (Feng, 2003 ▸), EKEQIU (Lin *et al.*, 2003 ▸), ESUZUN (Ling & Feng, 2003 ▸) and GAVZAF (Zheng *et al.*, 2005 ▸)]. Organometallic coordination compounds based on benzimidazole occur with various *d*-block metals, as well as with derivatives of this ligand, for example XUGJIW (Siddikova *et al.*, 2024 ▸); additionally, organic salts involving benzimidazole have been reported, *e.g.* HOWKUD (Mukhammadiev *et al.*, 2024 ▸).

## 6. Synthesis and crystallization

Preparation of solutions: (*a*) CoCl_2_·6H_2_O (0.1 mmol) dissolved in 5 ml of ethanol, (*b*) BI (0.2 mmol) dissolved in 3 ml of ethanol, and (*c*) BICA (0.2 mmol) dissolved in 3 ml of ethanol. Solution (*a*) was added to solution (*b*) and stirred for 30 minutes at room temperature on a magnetic stirrer. After that, solution (*c*) was added dropwise and the mixture was stirred for 12 h, during which it acquired a pink color. After several days a pale-pink precipitate formed, which was filtered off and washed several times with ethanol. Because both the initial precipitate and the obtained crystals were soluble in *N*,*N*-dimethylformamide (DMF) and dimethyl sulfoxide (DMSO), recrystallization was carried out from *N*,*N*-dimethylformamide (DMF). Bright-pink single crystals were obtained after recrystallization.
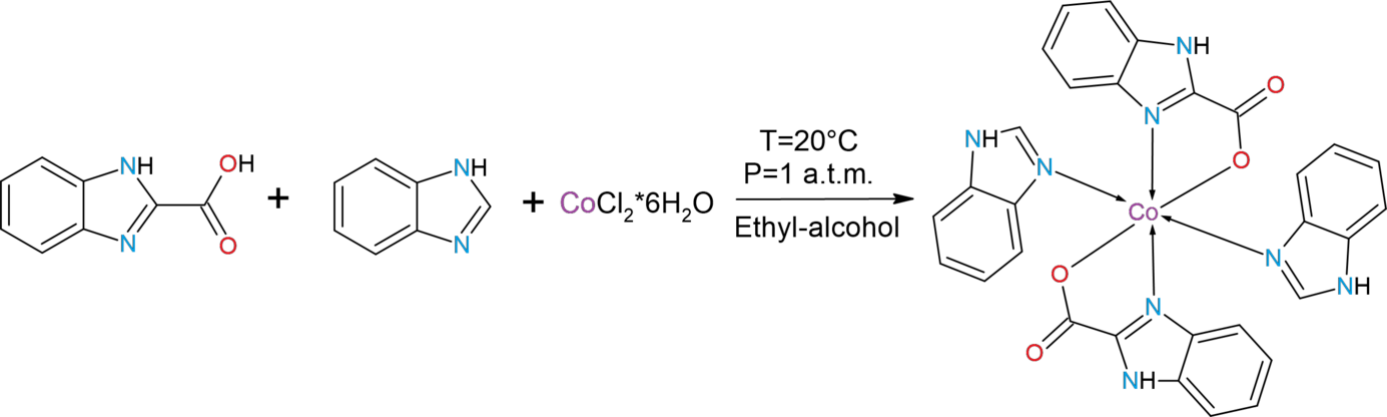


## 7. Refinement

Crystallographic data, data-collection conditions and structure-refinement parameters are summarized in Table 3 ▸. Hydrogen atoms were placed in calculated idealized positions and refined using a riding model with C—H distances of 0.93–0.98 Å and *U*_iso_(H) = 1.2*U*_eq_(C).

## Supplementary Material

Crystal structure: contains datablock(s) I. DOI: 10.1107/S2056989025011211/jp2023sup1.cif

Structure factors: contains datablock(s) I. DOI: 10.1107/S2056989025011211/jp2023Isup2.hkl

CCDC reference: 2515503

Additional supporting information:  crystallographic information; 3D view; checkCIF report

## Figures and Tables

**Table 1:** Selected geometric parameters (Å, °)

Co1—O1	2.128 (4)	Co1—N1	2.101 (5)
Co1—N3	2.162 (5)		
			
N1—Co1—O1	79.00 (18)	N1—Co1—N3	88.9 (2)

**Table 2:** Hydrogen-bond geometry (Å, °) *Cg* is the centroid of the C3–C8ring.

*D*—H⋯*A*	*D*—H	H⋯*A*	*D*⋯*A*	*D*—H⋯*A*
N2—H2⋯O2^ii^	0.86 (1)	1.91 (1)	2.750 (8)	167 (1)
N4—H4⋯O1^iii^	0.86 (1)	2.41 (1)	3.044 (8)	132 (1)
N4—H4⋯O2^iii^	0.86 (1)	2.30 (1)	3.064 (8)	148 (1)
C12—H12⋯*Cg*5^iv^	0.93 (2)	2.83 (2)	3.702 (15)	156 (2)

Symmetry codes: (ii) 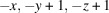; (iii) 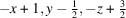; (iv) 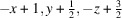.

**Table 3:** Experimental details

Crystal data	
Chemical formula	[Co(C_8_H_5_N_2_O_2_)_2_(C_7_H_6_N_2_)_2_]
*M*_r_	617.49
Crystal system, space group	Monoclinic, *P*2_1_/*c*
Temperature (K)	273
*a*, *b*, *c* (Å)	10.449 (4), 13.111 (5), 11.508 (5)
β (°)	113.427 (17)
*V* (Å^3^)	1446.6 (11)
*Z*	2
Radiation type	Mo *K*α
μ (mm^−1^)	0.64
Crystal size (mm)	0.42 × 0.28 × 0.08

Data collection	
Diffractometer	Bruker APEXII CCD
Absorption correction	Multi-scan (*SADABS*; Krause *et al.*, 2015 ▸)
*T*_min_, *T*_max_	0.672, 0.754
No. of measured, independent and observed [*I* ≥ 2u(*I*)] reflections	3327, 3324, 1747
*R*_int_	0.050
(sin θ/λ)_max_ (Å^−1^)	0.651

Refinement	
*R*[*F*^2^ > 2σ(*F*^2^)], *wR*(*F*^2^), *S*	0.119, 0.337, 1.01
No. of reflections	3324
No. of parameters	196
No. of restraints	120
H-atom treatment	H atoms treated by a mixture of independent and constrained refinement
Δρ_max_, Δρ_min_ (e Å^−3^)	2.22, −1.92

Computer programs: *APEX2* and *SAINT* (Bruker, 2014 ▸), *OLEX2.refine* (Bourhis *et al.*, 2015 ▸), *OLEX2* (Dolomanov *et al.*, 2009 ▸), *Mercury* (Macrae *et al.*, 2020 ▸), *ShelXle* (Hübschle *et al.*, 2011 ▸) and *PLATON* (Spek, 2020 ▸).

## supplementary crystallographic information

### Bis(1H-benzimidazole-κN3)bis(benzimidazole-2-carboxylato-κ2N3,O)cobalt(II) Crystal data

**Table:**

[Co(C_8_H_5_N_2_O_2_)_2_(C_7_H_6_N_2_)_2_]	*F*(000) = 634
*M**_r_* = 617.49	*D*_x_ = 1.418 Mg m^−^^3^
Monoclinic, *P*2_1_/*c*	Mo *K*α radiation, λ = 0.71073 Å
*a* = 10.449 (4) Å	Cell parameters from 9790 reflections
*b* = 13.111 (5) Å	θ = 2.2–22.5°
*c* = 11.508 (5) Å	µ = 0.64 mm^−^^1^
β = 113.427 (17)°	*T* = 273 K
*V* = 1446.6 (11) Å^3^	Plate, clear light pink
*Z* = 2	0.42 × 0.28 × 0.08 mm

### Bis(1H-benzimidazole-κN3)bis(benzimidazole-2-carboxylato-κ2N3,O)cobalt(II) Data collection

**Table:**

Bruker APEXII CCD diffractometer	1747 reflections with *I*≥ 2u(*I*)
ω scans	*R*_int_ = 0.050
Absorption correction: multi-scan (SADABS; Krause *et al.*, 2015)	θ_max_ = 27.6°, θ_min_ = 2.6°
*T*_min_ = 0.672, *T*_max_ = 0.754	*h* = −13→12
3327 measured reflections	*k* = 0→17
3324 independent reflections	*l* = 0→14

### Bis(1H-benzimidazole-κN3)bis(benzimidazole-2-carboxylato-κ2N3,O)cobalt(II) Refinement

**Table:**

Refinement on *F*^2^	22 constraints
Least-squares matrix: full	Primary atom site location: dual
*R*[*F*^2^ > 2σ(*F*^2^)] = 0.119	Hydrogen site location: inferred from neighbouring sites
*wR*(*F*^2^) = 0.337	H atoms treated by a mixture of independent and constrained refinement
*S* = 1.01	*w* = 1/[σ^2^(*F*_o_^2^) + (0.1973*P*)^2^] where *P* = (*F*_o_^2^ + 2*F*_c_^2^)/3
3324 reflections	(Δ/σ)_max_ = −0.0003
196 parameters	Δρ_max_ = 2.22 e Å^−^^3^
120 restraints	Δρ_min_ = −1.92 e Å^−^^3^

### Bis(1H-benzimidazole-κN3)bis(benzimidazole-2-carboxylato-κ2N3,O)cobalt(II) Fractional atomic coordinates and isotropic or equivalent isotropic displacement parameters (Å^2^)

**Table:**

	*x*	*y*	*z*	*U*_iso_*/*U*_eq_	
Co1	0.5	0.5	0.5	0.0425 (5)	
O2	0.1616 (5)	0.5781 (3)	0.5794 (5)	0.0611 (13)	
O1	0.3716 (4)	0.5810 (3)	0.5730 (4)	0.0492 (11)	
N2	0.1003 (5)	0.3967 (4)	0.4220 (5)	0.0523 (14)	
H2	0.0250 (5)	0.4102 (4)	0.4323 (5)	0.0628 (17)*	
N3	0.5820 (5)	0.4044 (4)	0.6666 (5)	0.0526 (14)	
N1	0.3189 (5)	0.4098 (4)	0.4329 (5)	0.0478 (14)	
C3	0.2586 (7)	0.3223 (5)	0.3667 (6)	0.0478 (16)	
C8	0.1262 (7)	0.3132 (5)	0.3579 (6)	0.0522 (17)	
C2	0.2511 (6)	0.5450 (5)	0.5456 (6)	0.0434 (15)	
N4	0.6677 (7)	0.2634 (5)	0.7808 (7)	0.075 (2)	
H4	0.6871 (7)	0.1999 (5)	0.7968 (7)	0.090 (2)*	
C1	0.2220 (6)	0.4517 (5)	0.4647 (6)	0.0423 (15)	
C15	0.6984 (10)	0.3401 (7)	0.8632 (8)	0.090 (3)	
C10	0.6416 (12)	0.4264 (6)	0.7911 (9)	0.098 (3)	
C7	0.0370 (8)	0.2328 (6)	0.2923 (7)	0.068 (2)	
H7	−0.0550 (8)	0.2272 (6)	0.2840 (7)	0.082 (3)*	
C5	0.2337 (9)	0.1699 (6)	0.2510 (7)	0.077 (2)	
H5	0.2686 (9)	0.1196 (6)	0.2145 (7)	0.092 (3)*	
C6	0.0995 (10)	0.1636 (6)	0.2418 (8)	0.086 (3)	
H6	0.0464 (10)	0.1083 (6)	0.1980 (8)	0.103 (3)*	
C9	0.5984 (9)	0.3079 (7)	0.6647 (8)	0.082 (3)	
H9	0.5656 (9)	0.2705 (7)	0.5898 (8)	0.099 (3)*	
C4	0.3154 (8)	0.2480 (6)	0.3125 (7)	0.067 (2)	
H4a	0.4068 (8)	0.2530 (6)	0.3191 (7)	0.081 (3)*	
C14	0.7669 (17)	0.3382 (9)	0.9911 (10)	0.165 (5)	
H14	0.8126 (17)	0.2808 (9)	1.0360 (10)	0.198 (6)*	
C11	0.640 (2)	0.5186 (9)	0.8471 (12)	0.167 (6)	
H11	0.601 (2)	0.5768 (9)	0.8000 (12)	0.201 (7)*	
C13	0.762 (2)	0.4299 (11)	1.0480 (14)	0.214 (7)	
H13	0.802 (2)	0.4327 (11)	1.1360 (14)	0.257 (8)*	
C12	0.701 (2)	0.5195 (11)	0.9814 (15)	0.216 (7)	
H12	0.701 (2)	0.5790 (11)	1.0253 (15)	0.260 (8)*	

### Bis(1H-benzimidazole-κN3)bis(benzimidazole-2-carboxylato-κ2N3,O)cobalt(II) Atomic displacement parameters (Å^2^)

**Table:**

	*U*^11^	*U*^22^	*U*^33^	*U*^12^	*U*^13^	*U*^23^
Co1	0.0417 (7)	0.0411 (8)	0.0423 (8)	−0.0004 (5)	0.0143 (5)	−0.0002 (5)
O2	0.049 (3)	0.064 (3)	0.070 (3)	0.005 (2)	0.023 (2)	−0.015 (3)
O1	0.045 (2)	0.051 (3)	0.044 (3)	−0.0022 (19)	0.009 (2)	−0.003 (2)
N2	0.045 (3)	0.053 (3)	0.053 (3)	−0.007 (2)	0.013 (3)	−0.006 (3)
N3	0.061 (3)	0.040 (3)	0.056 (4)	−0.001 (2)	0.022 (3)	0.003 (3)
N1	0.046 (3)	0.055 (3)	0.040 (3)	0.006 (2)	0.015 (2)	−0.007 (3)
C3	0.045 (4)	0.051 (4)	0.038 (4)	0.004 (3)	0.006 (3)	−0.005 (3)
C8	0.055 (4)	0.049 (4)	0.042 (4)	−0.008 (3)	0.008 (3)	−0.002 (3)
C2	0.043 (3)	0.041 (4)	0.039 (4)	0.009 (3)	0.009 (3)	0.002 (3)
N4	0.091 (5)	0.056 (4)	0.071 (5)	0.011 (3)	0.024 (4)	0.023 (4)
C1	0.033 (3)	0.050 (4)	0.037 (4)	0.000 (3)	0.007 (3)	0.006 (3)
C15	0.130 (7)	0.070 (5)	0.044 (4)	−0.017 (4)	0.007 (4)	0.014 (3)
C10	0.164 (9)	0.058 (5)	0.053 (5)	0.001 (4)	0.023 (5)	0.006 (3)
C7	0.073 (5)	0.064 (5)	0.049 (4)	−0.014 (4)	0.005 (4)	−0.016 (4)
C5	0.080 (6)	0.068 (5)	0.067 (6)	0.004 (4)	0.014 (5)	−0.025 (4)
C6	0.093 (7)	0.064 (5)	0.065 (6)	−0.022 (4)	−0.006 (5)	−0.024 (4)
C9	0.109 (7)	0.068 (6)	0.054 (5)	−0.011 (5)	0.015 (5)	0.000 (4)
C4	0.074 (5)	0.072 (5)	0.051 (5)	0.003 (4)	0.019 (4)	−0.011 (4)
C14	0.280 (13)	0.108 (7)	0.050 (5)	−0.008 (6)	0.005 (5)	0.007 (4)
C11	0.324 (16)	0.087 (6)	0.069 (6)	0.015 (6)	0.054 (7)	−0.008 (4)
C13	0.391 (17)	0.132 (9)	0.073 (7)	0.010 (7)	0.043 (7)	−0.003 (5)
C12	0.411 (19)	0.127 (8)	0.082 (7)	0.028 (8)	0.068 (7)	−0.009 (5)

### Bis(1H-benzimidazole-κN3)bis(benzimidazole-2-carboxylato-κ2N3,O)cobalt(II) Geometric parameters (Å, º)

**Table:**

Co1—O1	2.128 (4)	N4—C15	1.331 (10)
Co1—O1^i^	2.128 (4)	N4—C9	1.372 (10)
Co1—N3	2.162 (5)	C15—C10	1.389 (12)
Co1—N3^i^	2.162 (5)	C15—C14	1.357 (12)
Co1—N1	2.101 (5)	C10—C11	1.373 (14)
Co1—N1^i^	2.101 (5)	C7—H7	0.9300
O2—C2	1.226 (7)	C7—C6	1.375 (12)
O1—C2	1.262 (7)	C5—H5	0.9300
N2—H2	0.8600	C5—C6	1.366 (11)
N2—C8	1.405 (8)	C5—C4	1.341 (10)
N2—C1	1.372 (7)	C6—H6	0.9300
N3—C10	1.346 (10)	C9—H9	0.9300
N3—C9	1.278 (9)	C4—H4a	0.9300
N1—C3	1.383 (8)	C14—H14	0.9300
N1—C1	1.324 (8)	C14—C13	1.379 (16)
C3—C8	1.351 (9)	C11—H11	0.9300
C3—C4	1.409 (10)	C11—C12	1.418 (18)
C8—C7	1.410 (9)	C13—H13	0.9300
C2—C1	1.494 (10)	C13—C12	1.41 (2)
N4—H4	0.8600	C12—H12	0.9300
			
O1^i^—Co1—O1	180.0	N1—C1—N2	112.6 (6)
N3^i^—Co1—O1^i^	91.66 (18)	C2—C1—N2	125.7 (5)
N3—Co1—O1	91.66 (18)	C2—C1—N1	121.6 (5)
N3^i^—Co1—O1	88.34 (18)	C10—C15—N4	105.4 (7)
N3—Co1—O1^i^	88.34 (18)	C14—C15—N4	129.3 (9)
N3—Co1—N3^i^	180.0	C14—C15—C10	125.3 (10)
N1^i^—Co1—O1	101.00 (18)	C15—C10—N3	111.6 (8)
N1^i^—Co1—O1^i^	79.00 (18)	C11—C10—N3	126.9 (9)
N1—Co1—O1^i^	101.00 (18)	C11—C10—C15	121.2 (10)
N1—Co1—O1	79.00 (18)	H7—C7—C8	123.3 (5)
N1—Co1—N3	88.9 (2)	C6—C7—C8	113.3 (8)
N1^i^—Co1—N3	91.1 (2)	C6—C7—H7	123.3 (5)
N1^i^—Co1—N3^i^	88.9 (2)	C6—C5—H5	119.8 (5)
N1—Co1—N3^i^	91.1 (2)	C4—C5—H5	119.8 (5)
N1^i^—Co1—N1	180.0	C4—C5—C6	120.4 (8)
C2—O1—Co1	115.9 (4)	C5—C6—C7	124.9 (7)
C8—N2—H2	127.7 (3)	H6—C6—C7	117.5 (5)
C1—N2—H2	127.7 (4)	H6—C6—C5	117.5 (5)
C1—N2—C8	104.7 (5)	N4—C9—N3	115.2 (8)
C10—N3—Co1^i^	132.2 (5)	H9—C9—N3	122.4 (5)
C9—N3—Co1^i^	124.5 (5)	H9—C9—N4	122.4 (5)
C9—N3—C10	102.8 (7)	C5—C4—C3	118.0 (8)
C3—N1—Co1^i^	144.7 (4)	H4a—C4—C3	121.0 (4)
C1—N1—Co1^i^	109.9 (4)	H4a—C4—C5	121.0 (5)
C1—N1—C3	105.4 (5)	H14—C14—C15	123.3 (7)
C8—C3—N1	110.1 (6)	C13—C14—C15	113.3 (12)
C4—C3—N1	129.6 (6)	C13—C14—H14	123.3 (8)
C4—C3—C8	120.3 (6)	H11—C11—C10	122.1 (7)
C3—C8—N2	107.3 (5)	C12—C11—C10	115.8 (12)
C7—C8—N2	129.7 (7)	C12—C11—H11	122.1 (8)
C7—C8—C3	123.0 (7)	H13—C13—C14	117.9 (8)
O1—C2—O2	126.8 (6)	C12—C13—C14	124.2 (14)
C1—C2—O2	119.7 (6)	C12—C13—H13	117.9 (9)
C1—C2—O1	113.5 (5)	C13—C12—C11	119.7 (14)
C15—N4—H4	127.5 (4)	H12—C12—C11	120.1 (8)
C9—N4—H4	127.5 (5)	H12—C12—C13	120.1 (9)
C9—N4—C15	104.9 (7)		
			
Co1—O1—C2—O2	178.5 (4)	C3—C8—N2—C1	0.6 (6)
Co1—O1—C2—C1	−0.7 (4)	C3—C8—C7—C6	1.8 (8)
Co1—N3—C10—C15	−170.5 (10)	C3—C4—C5—C6	0.2 (9)
Co1—N3—C10—C11	14.8 (12)	C8—N2—C1—C2	−176.1 (5)
Co1—N3—C9—N4	172.3 (6)	C8—C3—N1—C1	0.8 (6)
Co1—N1—C3—C8	−178.7 (8)	C8—C3—C4—C5	1.0 (8)
Co1—N1—C3—C4	1.4 (9)	C8—C7—C6—C5	−0.7 (9)
Co1—N1—C1—N2	179.3 (4)	N4—C15—C10—C11	172.7 (11)
Co1—N1—C1—C2	−4.5 (5)	N4—C15—C14—C13	−171.0 (17)
O2—C2—C1—N2	0.1 (7)	N4—C9—N3—C10	−1.0 (9)
O2—C2—C1—N1	−175.6 (6)	C1—N2—C8—C7	−178.3 (5)
O1—C2—C1—N2	179.4 (5)	C1—N1—C3—C4	−179.0 (5)
O1—C2—C1—N1	3.7 (6)	C15—C10—N3—C9	2.0 (10)
N2—C8—C3—N1	−0.9 (6)	C15—C10—C11—C12	0.7 (16)
N2—C8—C3—C4	179.0 (5)	C15—C14—C13—C12	−4 (2)
N2—C8—C7—C6	−179.5 (8)	C10—C15—N4—C9	1.6 (10)
N2—C1—N1—C3	−0.4 (6)	C10—C15—C14—C13	7.2 (15)
N3—C10—C15—N4	−2.4 (10)	C10—C11—C12—C13	2 (2)
N3—C10—C15—C14	179.0 (11)	C7—C8—C3—C4	−2.0 (8)
N3—C10—C11—C12	175.0 (18)	C7—C6—C5—C4	−0.3 (11)
N3—C9—N4—C15	−0.5 (9)	C9—N3—C10—C11	−172.7 (12)
N1—C3—C8—C7	178.1 (5)	C9—N4—C15—C14	−179.9 (11)
N1—C3—C4—C5	−179.2 (8)	C14—C15—C10—C11	−5.8 (17)
N1—C1—N2—C8	−0.1 (6)	C14—C13—C12—C11	−0 (3)
C3—N1—C1—C2	175.8 (5)		

Symmetry code: (i) −*x*+1, −*y*+1, −*z*+1.

### Bis(1H-benzimidazole-κN3)bis(benzimidazole-2-carboxylato-κ2N3,O)cobalt(II) Hydrogen-bond geometry (Å, º)

*Cg* is the centroid of the C3–C8ring.

**Table:**

*D*—H···*A*	*D*—H	H···*A*	*D*···*A*	*D*—H···*A*
N2—H2···O2^ii^	0.86 (1)	1.91 (1)	2.750 (8)	167 (1)
N4—H4···O1^iii^	0.86 (1)	2.40 (1)	3.044 (8)	132 (1)
N4—H4···O2^iii^	0.86 (1)	2.30 (1)	3.064 (8)	148 (1)
C12—H12···*Cg*5^iv^	0.93 (2)	2.83 (2)	3.702 (15)	156 (2)

Symmetry codes: (ii) −*x*, −*y*+1, −*z*+1; (iii) −*x*+1, *y*−1/2, −*z*+3/2; (iv) −*x*+1, *y*+1/2, −*z*+3/2.
